# Using linkage to assess coverage of population estimates.

**DOI:** 10.23889/ijpds.v7i3.2038

**Published:** 2022-08-25

**Authors:** Sarah Collyer, Josie Plachta

**Affiliations:** 1 Office for National Statistics

## Objectives

The Demographic Index (DI) comprises of five linked administrative datasets, used for population estimation. Current linkage methods are not ideal to utilise the power of this asset. Using the 2021 England and Wales Census, we are developing an innovative composite linkage method to fully utilise the power of the DI.

## Approach

Using non-greedy deterministic and probabilistic linkage methods, we will link the DI to the Census at a composite level where we believe links exist – i.e., linking a Census cluster (consisting of linked Census and Census Coverage Survey (CCS) records) with a DI cluster (consisting of linked records from the data sources used to make the DI). We will then conduct a pairwise linkage of records from these linked clusters to link individual source records to the Census. We will utilise clerical review to resolve uncertain and conflicting links and to inform the quality of our linkage.

## Results

We anticipate producing a high-quality linkage that will inform how the coverage of the DI compares to Census (through the composite-level linkage) and the quality of the DI itself (through the pairwise-level linkage). We have developed a clerical matching system that can display composite-level linkage, i.e., candidate cluster-pairs. We will tailor our clerical review and quality assessment to records that fall within carefully chosen postcode areas, to ensure all hard-to-count groups and geographical areas are sampled. Working with large datasets is a challenge we are overcoming by using distributed computing and search space reduction.

The 2021 Census has been previously linked to the CCS with high accuracy; these records are considered intrinsically linked.

## Conclusion

To assess national population estimates’ quality and the policy decisions based upon them, we are linking a key composite population-level dataset to the 2021 England and Wales Census. The presentation will showcase the methods we are developing and how we are ensuring the highest quality possible.

